# Selected coffee (*Coffea arabica* L.) extracts inhibit intestinal α-glucosidases activities *in-vitro* and postprandial hyperglycemia in SD Rats

**DOI:** 10.1186/s12906-022-03726-7

**Published:** 2022-09-23

**Authors:** Haimanot Mitiku, Tae Yang Kim, Hanna Kang, Emmanouil Apostolidis, Jung-Yun Lee, Young-In Kwon

**Affiliations:** 1 Department of Food and Nutrition, Hannam University, Daejeon, 34054 Korea; 2 Department of Chemistry and Food Science, Framingham State University, Framingham, MA 01701 USA

**Keywords:** α-glucosidases, α-amylase hyperglycemia, Coffee arabica, Inhibition, Diabetes

## Abstract

One of the therapeutic approaches for decreasing postprandial hyperglycemia is to retard absorption of glucose by the inhibition of carbohydrate hydrolyzing enzymes, α-amylase, and α-glucosidases, in the digestive organs. Coffee consumption has been reported to beneficial effects for controlling calorie and cardiovascular diseases, however, the clear efficacy and mode of action are yet to be proved well. Therefore, in this study we evaluated *in- vitro* rat intestinal α-glucosidases and porcine α-amylase inhibitory activities as well as *in vivo* (Sprague–Dawley rat model) blood glucose lowering effects of selected coffee extracts. The water extracted Sumatra coffee (SWE) showed strong α-glucosidase inhibitory activity (IC_50_, 4.39 mg/mL) in a dose-dependent manner followed by Ethiopian water extract (EWE) (IC_50_, 4.97) and Guatemala water extract (GWE) (IC_50_, 5.19). Excepted for GWE all the coffee types significantly reduced the plasma glucose level at 0.5 h after oral intake (0.5 g/kg-body weight) in sucrose and starch-loaded SD rats. In sucrose loading test SWE (*p* < 0.001) and EWE (*p* < 0.05) had significantly postprandial blood glucose reduction effect, when compared to control. The maximum blood glucose levels (C*max*) of EWE administration group were decreased by about 18% (from 222.3 ± 16.0 to 182.5 ± 15.4, *p* < 0.01) and 19% (from 236.2 ± 25.1 to 191.3 ± 13.2 h·mg/dL, *p* < 0.01) in sucrose and starch loading tests, respectively. These results indicate that selected coffee extract may improve exaggerated postprandial spikes in blood glucose via inhibition of intestinal sucrase and thus delays carbohydrate absorption. These *in vitro* and *in vivo* studies therefore could provide the biochemical rationale for the benefit of coffee-based dietary supplement and the basis for further clinical study.

## Introduction

Recent studies showed that more than 463 million people around the world are affected by Type 2 Diabetes (T2D) in 2019 and in 2045 there will be more than 700 million people with diabetes worldwide [1]. Non-insulin-dependent diabetes mellitus (NIDDM) is a metabolic disorder characterized by hyperglycemia with resistance to ketosis [2]. Diabetes mellitus is a disease of excess glucose in the plasma, qualitative and quantitative abnor-malities of carbohydrate and lipid metabolism, characteristic pathological changes in nerves and small blood vessels, and intensification of atherosclerosis [2] and is characterized by either a total or partial loss of insulin secretion and/or resistance to insulin action leading to a chronic state of hyperglycemia in the blood [3, 4]. Microvascular complication, such as retinopathy that could lead to blindness, or nephropathy, is common in poor glycemic control patients. Furthermore, an increased risk of cardiovascular disease is obvious in T2D patients, which is attributable to endothelial dysfunction [5, 6].

Hyperglycemia, a rapid rise in blood glucose levels in NIDDM patients occurs when the multiple homeostatic mechanisms that minimize glucose fluctuations and restore normal glucose levels following a meal are blunted due to hydrolysis of starch by pancreatic α-amylase and subsequent absorption of glucose in the small intestine by α-glucosidases such as sucrose, maltase, and glucoamylase [7, 8]. These enzymes play a pivotal role in the final stage of carbohydrate digestion and one of the useful approaches to reduce post-prandial hyperglycemia is to retard the absorption of glucose by inhibition of carbohydrate hydrolyzing enzymes, such as α-glucosidase and α-amylase, in the digestive organs [3, 7, 9, 10].

The necessity of pharmacology therapy for the proper control of hyperglycemia has been emphasized [11] though other natural treatments have significant importance. Drugs that inhibit carbohydrate hydrolyzing enzymes for the management of type 2 diabetes include acarbose, voglibose® and miglitol® [12]. However, these drugs carry undesired side effects such as abdominal distension, flatulence, meteorism, and possibly diarrhea [10]. Therefore, the attention of many researchers has been directed towards searching for natural inhibitors with no or very minimal side effects.

Coffee is among the widely consumed and one of the most popular beverages of the world brewed from roasted coffee beans [13, 14] with approximately 500 billion cups of coffee being consumed worldwide annually [15]. And it is the most important food commodity globally and ranks second, next to crude oil, among all commodities [16]. Next to fuel it’s also the most traded [17] and globally it is the most consumed functional food. The coffee beverage is known for its stimulating properties attributed mainly to caffeine; however, coffee contains a large number of chemical compounds with many biologically active properties, including phenolic compounds [18].

The health benefits of coffee consumption have been well discussed [19]. Several epidemiological studies also reported that coffee consumption may reduce the risk of chronic diseases such as T2D [12], Alzheimer’s disease and Parkinson’s disease [20, 21], and liver disease [22]. The disease-reducing potential may be associated with the phenolic compounds found in coffee [23]. The phenolic compounds found in green bean includes chlorogenic acids (CGA), caffeic, ferulic and dimethoxycinnamic acids, conjugation of hydroxycinnamic acids with amino acids (cinnamoyl amides) or glycosides (cinnamoyl glycosides). CGA, an ester of caffeic acid has been reported to be the main component of coffee [24]. Coffee beans are also alleged to be a good dietary source of CGA [25]. The anti-diabetic potential of coffee through its phenolic constituents such as chlorogenic acid caffeic acid and diterpenes in animal models and clinical trials has been reported [24, 26]. However, little is known on the effect of different coffee varieties on the rat intestinal α-glucosidase inhibitory activity in *in-vitro* and *in-vivo* animal experiments.

Thus, in this study we extracted Sumatra, Guatemala, and Ethiopia coffee using distilled water (DW) and ethanol and then the following studies were conducted: (i) to measure total phenolic content (ii) to compare the *in-vitro* inhibitory activities on α-glucosidases and α-amylase (iii) to conduct *in-vivo* animal study to investigate the effect of the water and ethanol extract coffees on postprandial glycemic response and compared their effects to a known pharmacological α-glucosidase inhibitor, acarbose, in sucrose-fed Sprague–Dawley rats.

## Materials and methods

### Materials

Guatemala, Ethiopia and Sumatra Medium, roasted commercial coffee (*Coffea arabica* L.) powders were purchased from the Folgers coffee company (Orrville, OH, USA). Porcine pancreatic α-amylase (EC 3.2.1.1), rat intestinal acetone powders of α-glucosidase (EC 3.2.1.20), and soluble starch (S9765-250G) were also purchased from Sigma-Aldrich Co. (St. Louis, MO, USA). Unless noted, all chemicals were purchased from Sigma-Aldrich Co. (St. Louis, MO, USA). The tested doses for the *in vitro* experiments described below were determined after initial screening. Following the initial screening we evaluated three doses that would yield to at least one inhibitory effect over 50% and one inhibitory effect below 50% (so IC_50_ values can be determined).

### Extraction

Sample extraction was conducted according to [27] with little modification. Ground coffee beans were subjected to two types of extraction methods namely, water and ethanol extraction methods. The ground roasted beans (each 50 g) were dissolved 500 mL of dis-tilled water and 70% ethanol. Water dissolved samples were boiled in autoclave adjusted to 121 °C for 3 h and then centrifuged to 15 min. Whereas ethanol extracted samples were stirred in an electrical shaker for 2 h at 40 °C. Water and ethanol dissolved sample solutions were filtered using a Büchner funnel and then percolated using Whatman no. 2 filter paper. The solvent from extracted samples was removed using a vacuum rotary evaporator under reduced pressure conditions in a water bath set at 60 °C. The concentrated samples were stored in a deep freezer set at -70 °C for 1 day and the remaining solvent was removed by freeze- drier. The dried samples were stored at -20 °C until use.

### Total phenolic content analysis

The coffee extracts' total polyphenol contents (TPCs) were analyzed using the Folin–Ciocalteau method following a method [7]. One mL of coffee extract was transferred into a test tube and mixed with 1 mL of distilled water or 95% ethanol, and 5 mL of distilled water. To each sample 0.5 mL of 50% (v/v) Folin-Ciocalteu reagent was added and mixed. After 5 min, 1 mL of 5% Na_2_CO_3_ was added to the reaction mixture and allowed to stand for 1 h. The absorbance was read at 725 nm using a spectrophotometer (UV-160A; Shimadzu Inc., Kyoto, Japan). The absorbance values were converted to total phenolics and were expressed in mg equivalents of gallic acid/mL of the sample. Standard curves were established using various concentrations of gallic acid in 95% ethanol.

### Rat small intestinal α-glucosidase inhibition assay

In order to investigate the inhibitory effect of coffee extracts on the absorption of glucose, rat intestinal α-glucosidase inhibitory activity was determined using the substrate *p*-nitrophenyl-α-D-glucopyranoside (*p*NPG) according to [28] with a slight modification. A total of 0.6 g of rat-intestinal acetone powder was suspended in 9 mL of 0.1 M sodium phosphate buffer 0.9%, and the suspension was sonicated for 12 times 30 s at 4 °C. It was then centrifuged (13,000 × g, 30 min, 4 °C), and the resulting supernatant was used for the assay. Sample solution (50 μL) and 0.1 M phosphate buffer (pH 6.9, 100 μL) containing rat intestinal α-glucosidase solution (1.0 U/mL) was incubated at 37 °C for 10 min. After the incubation, 5 mM *p*NPG solution (50 μL) in 0.1 M phosphate buffer (pH 6.9) was added to each well at timed intervals. The reaction mixtures were further incubated at 37 °C for 30 min. Absorbance was measured at 405 nm and compared to a control that had 50 μL of buffer solution in place of the extract by Micro-Plate Reader (SpectraMAx® i3, Molecular devices LLC., Wals, Austria). The rat α-glucosidase inhibitory activity was expressed as percent inhibition and was calculated as follows:

$$\mathrm{Inhibition}\left(\mathrm{\%}\right)=\left[\frac{\Delta {A}_{\mathrm{Control }405}-\Delta {A}_{\mathrm{Extract}405}}{\Delta {A}_{\mathrm{Control }405}}\right]\times 100$$

### Porcine α-amylase inhibition assay

Porcine pancreatic α-amylase assay was conducted based on the method to [29] with slight modification. Porcine pancreatic α-amylase (EC 3.2.1.1) was purchased from Sigma Chemical Co. Sample solution (200 μL) and 0.02 M sodium phosphate buffer (pH 6.9 with 0.006 M sodium chloride, 500 μL) containing α-amylase solution (0.5 mg/mL, 15U/mL) were incubated at 25 °C for 10 min. After pre-incubation, 500 μL of a 1% starch solution in 0.02 M sodium phosphate buffer was added. The reaction mixture was then incubated at 25 °C for 10 min. The reaction was stopped with 1.0 mL of dinitrosalicylic acid (DNS). The reaction mixture was then incubated in a boiling water bath for 5 min and cooled to room temperature. The reaction mixture was then diluted after adding 1.0 mL distilled water, and absorbance was measured at 540 nm with micro-plate reader (SpectraMAx® i3; Molecular Devices LLC., Wals, Austria).

### Sucrase, maltase and glucoamylase inhibition assay

Sucrase, Maltase, and Glucoamylase Inhibition assay was performed by a method described in [3] The crude enzyme solution prepared from rat intestinal acetone powder was used as the small intestinal maltase, sucrase, and glucoamylase. Rat intestinal ace-tone powder (600 mg) was suspended in 9 mL of 0.1 M sodium phosphate buffer, and the suspension was sonicated 12 times for 30 s at 4 °C. After centrifugation (10,000 × g, 30 min, 4 °C), the resulting supernatant was used for the assay. The inhibitory activity was deter-mined by incubating a solution of an enzyme (100 μL), 0.1 M phosphate buffer (pH 7.0, 50 μL) containing 50μL of 36 and 68 mg/mL maltose and sucrose respectively, or 1% soluble starch, and a solution (50 μL) with various concentrations of sample solution. In the reaction mixture 200 μL of 12 N of H_2_SO_4_ was added to stop the reaction, and then the amount of liberated glucose was measured by the glucose oxidase method. The inhibitory activity was calculated from the formula as follows.

$$\mathrm{Inhibition}\left(\mathrm{\%}\right)=\left[\frac{\Delta {A}_{\mathrm{Control }530}-\Delta {A}_{\mathrm{Extract}530}}{\Delta {A}_{\mathrm{Control }530}}\right]\times 100$$

### *In-vivo* animal model

All animal procedures were approved by the Institutional Animal Care and Use Com-mittee (IACUC) of Hannam University (Approval number: HNU2016-0015–1). This study is reported in accordance with ARRIVE guidelines (https://arriveguidelines.org). Four-week-old male Sprague–Dawley (SD) rats were purchased from Joongang Experimental Animal Co. (Seoul, Korea) and fed with a standard diet (Samyang Diet Co., Seoul, Korea) and with water ad libitum for one week. Effect on hyperglycemia-induced by carbohydrate loads in Sprague–Dawley (SD) rats was determined by the inhibitory action of coffee extracts on postprandial hyperglycemia as described in [30]. The rats were housed in a ventilated room at 25 ± 2 °C with 50 ± 7% relative humidity and under an alternating 12-h light/dark cycle. After 6 groups of 5 male SD rats (180 ~ 200 g) were fasted for 24 h, 2.0 g/kg body weight of sucrose or starch were orally administrated concurrently with the control group (no treatment) and a known pharmacological α-glucosidase inhibitor, acarbose (5 mg/kg body weight) as a positive control. In the case of the administration group, administration samples were prepared by mixing starch or sugar with coffee extracts by dose in advance were orally administrated using a zonde injection needle. Blood glucose levels were measured by drawing blood from the tail (at 0, 0.5, 1, 2, and 3 h) and were determined by using the glucose oxidase method. These results were compared to control that did not receive and treatment.

### Blood analysis

The blood samples were then taken from the tail after administration and blood glucose levels were measured at 0, 0.5, 1, 2, and 3 h. The glucose level in blood was determined by the glucose oxidase method and compared with that of the control group. The parameters for blood glucose levels will be calculated Maximum observed peak blood glucose level (C_*max*_) and the time at which it is observed (T_*max*_) were determined based on the observed data. The area under the blood glucose-time curve up to the last sampled time-point (AUC_*last*_) was estimated by the trapezoidal rule.

### Statistical analysis

All data are presented as mean ± S.D. Statistical analyses were carried out using the statistical packages SPSS 11 (Statistical Package for Social Science 11, SPSS Inc., Chicago, IL, USA) program and the significance of each group was verified with the analyses of one-way ANOVA followed by Duncan’s test of *p* < 0.05. In addition, statistical significances in the animal study were determined by Student’s *t*-test (*p < 0.05; **p < 0.01; and *** *p* < 0.001). IC_50_ value was obtained from dose response curve of percent viability versus test concentrations. IC_50_ calculations were performed by using linear regression analysis. ED50plus v1.0 in Excel program.

## Results and discussion

### Total phenolic contents of the selected coffee extracts

**Fig. 1:**
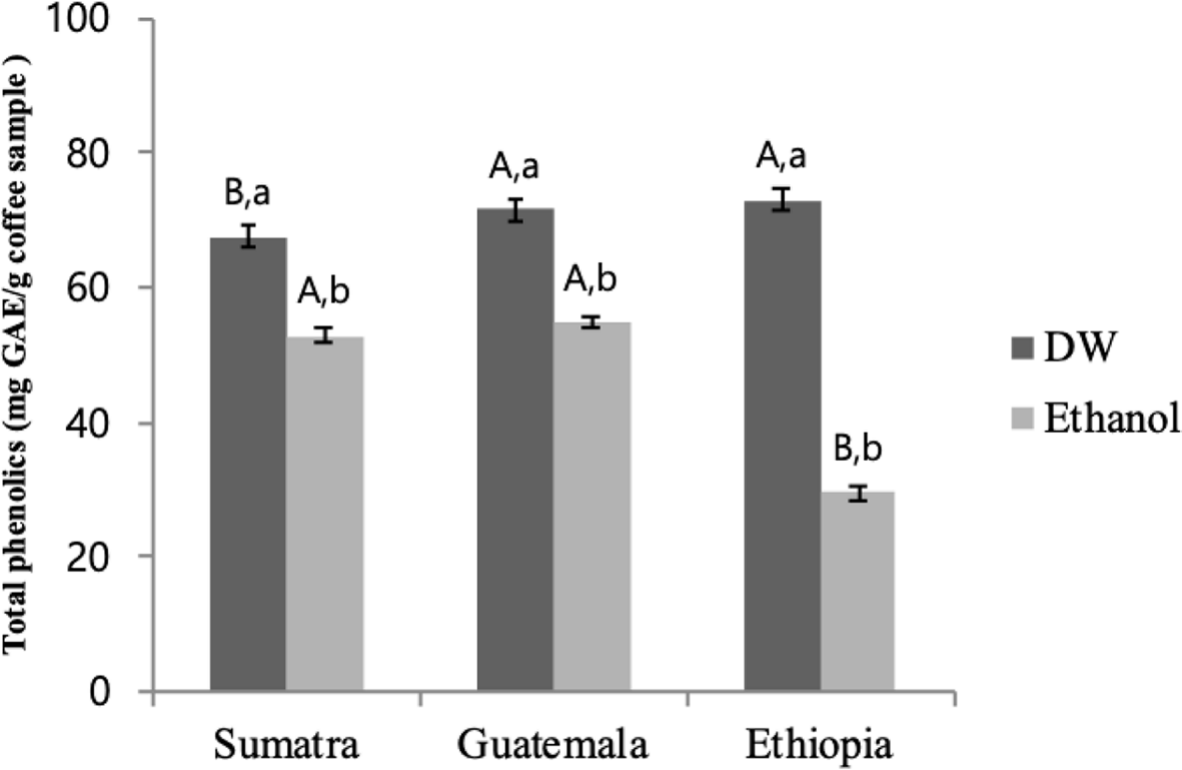
Total phenolic content (mg GAE/g sample) of water (DW) and ethyl alcohol (Ethanol) extracts of coffee variety. The results represent the mean ± S.D. Different uppercase letters indicate significant differences among different samples within same extraction method whereas dif-ferent lowercase letters indicate significant differences among different extraction method within same sample at *p* < 0.05 by Duncan’s multiple range test

The total phenolic contents (TPC) of selected coffee extracts were analyzed using the Folin-Ciocalteu method. Figure 1 shows the TPC of the tested water and ethanol coffee extracts. The highest average TPC value from water extract samples was observed in Sumatra coffee (67.65 ± 1.59 mg GAE/100 g) followed by Guatemala (71.58 ± 1.55) and Ethiopia (73.00 ± 0.57). In the case of coffee extracted by ethanol the higher value of TPC was recorded for Guatemala coffee with Sumatra and the lowest value was recorded from Ethiopia.

An excess of free radicals in the body is one of the causes of lifestyle diseases such as cancer or diseases of the circulatory system [14]. Therefore, it is essential that the human diet contains, among other nutrients, phenolic compounds. phenolic compounds have a number of beneficial health properties related to their potent antioxidant activity as well as hepatoprotective, hypoglycemic, and antiviral activities [23]. It has been reported that coffee is one of the dietary sources of phenolic compounds [24]. Also, it is reported that plant variety, species and growing/harvesting conditions can affect phenolic content in plants [31].

### α-glucosidase inhibitory activity

The α-glucosidases inhibitors, which interfere with enzymatic action in the brush-border of the small intestine, could slow the liberation of D-glucose from oligosaccharides and disaccharides resulting in reduced postprandial plasma glucose levels [28].

**Fig. 2:**
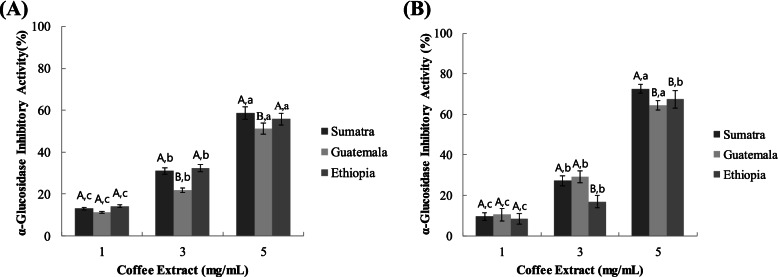
Dose dependent changes in rat intestinal α-glucosidase inhibitory activity (%inhibition) of water extracted coffee (**A**) and ethyl alcohol extracted coffee (**B**) types. Different uppercase letters indicate significant differences among different samples within same concentration whereas different lowercase letters indicate significant differences among different concentration within same sample at *p* < 0.05 by Duncan’s multiple range test

α-Glucosidase inhibitory activities of water and ethanol extracts of coffee samples are listed in Fig. 2A and B.

In water extracts, the higher α-glucosidase inhibitory activity was obtained from SWE (4.39 mg/mL of IC_50_) followed by EWE (4.97 mg/mL of IC_50_) and GWE (5.19 mg/mL of IC_50_) (Table 1).

**Table 1:** The half-maximal inhibitory concentration (IC_50_) values for rat intestinal α-glucosidase and pancreatic α-amylase by water extracted coffee types

		**IC**_**50**_** (mg/mL)**			
		**SWE**	**GWE**	**EWE**	**Acarbose®**
**α-glucosidase**	Water	4.39	> 5.00	4.97	< 0.50
	Ethanol	2.97	3.19	3.27	
**α-amylase**	Waster	> 5.00	> 5.00	> 5.00	< 0.50
	Ethanol	> 5.00	> 5.00	> 5.00	

The dose-dependent α-glucosidase inhibitory activity water extracted coffee samples were observed in Fig. 2A. At 5 mg/mL, SWE showed the higher α-glucosidase inhibitory activity (4.39 mg/mL of IC_50_); followed by EWE and GWE; however, there was no significant difference between SWE and EWE.

In similar pattern ethanol extracted coffee showed α-glucosidase inhibitory activity in a dose-dependent manner. SEE showed higher α-glucosidase inhibitory activity at higher concentrations followed by GEE and EEE (Fig. 2B). Table 1 showed that the IC_50_ (mg/mL) values of ethyl alcohol extracted coffee.

### α-amylase inhibition assay

The α-amylase inhibitors, which interfere with enzymatic action in the small intestine, could slow the liberation of maltose from starch, resulting in delaying maltose conversion to glucose and decreasing postprandial plasma glucose levels [30]. In our case, little or no inhibition of α-amylase was observed by coffee extracts (Table 1) linked to the side-effect due to increase non-digested starch in large intestine. reported that chlorogenic acid and phenolic acid from coffee are very weak inhibitors of human salivary α-amylase [32].

### Sucrase, maltase, and glucoamylase inhibition assay

**Fig. 3:**
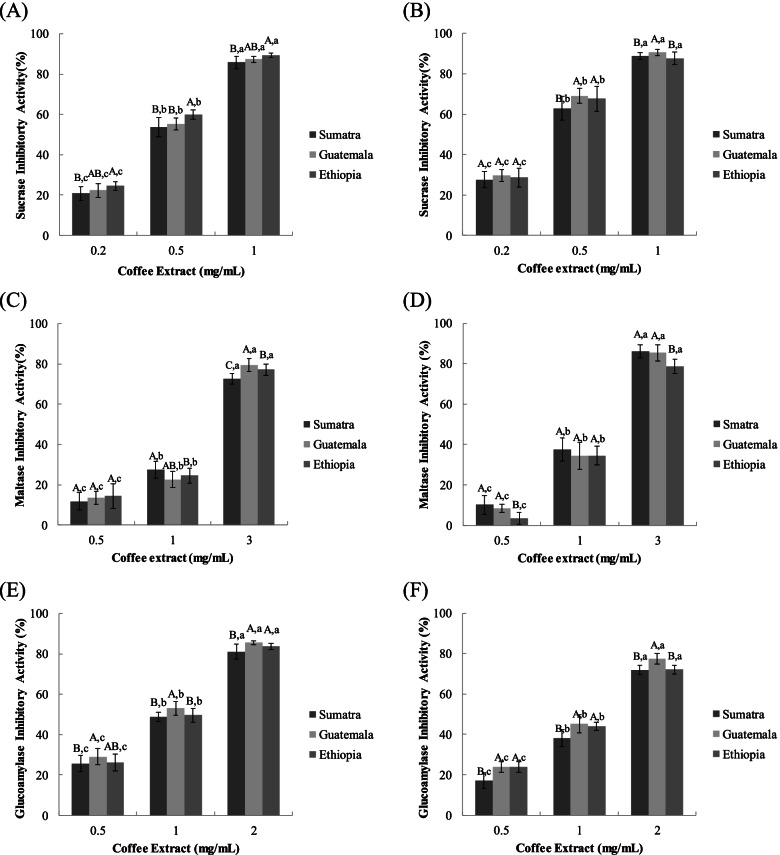
Dose dependent changes in rat intestinal sucrase, maltase and glucoamylase in-hibitory activity (% inhibition) of water extracted coffee (**A**, **C**, and **E**, respectively) and ethyl alcohol extracted coffee (Fig. **B**, **D**, and **F**) types The results represent the mean ± S.D. Different uppercase letters indicate significant differences among different samples within same concentration whereas different lowercase letters indicate significant differences among different concentration within same sample at *p* < 0.05 by Duncan’s multiple range test

It has been reported that most yeast α-glucosidase inhibitors did not show significant activities against mammalian α-glucosidase, due to the difference in molecular recognition in the target binding site of these enzymes. Therefore, rat small intestinal sucrase, maltase, and glucoamylase, the key α-glucosidases that catalyze the hydrolysis of disaccharides to glucose were used for estimating the inhibitory activities of coffee extracts [33]. To determine the specificity of the observed inhibitory activity, we examined the effect of coffee extracts of all three coffee types on rat small intestinal sucrase, maltase, and glucoamylase. Both water and ethanol coffee extracts showed intestinal sucrase inhibitory activity in a dose-dependent manner. Water extracted coffee EWE showed higher inhibitory activity in all concentrations followed by SWE and GWE (Fig. 3A). In ethanol extracts, SEE showed higher inhibitory activity than EEE and GEE (Fig. 3B). At 3 mg/mL concentrations GWE showed higher maltase inhibitory activity in water extracts followed by EWE and EWE whereas in ethanol extracts SEE showed maltase inhibitory activity in a similar percentage with GEE but significantly higher than EEE (Figs. 3C and D). For glucoamylase, a higher inhibitory percentage was obtained by GWE in water extracts and GEE in ethanol extracts and showed in both water and ethanol extracts (Figs. 3E and F).

The IC_50_ value of sucrase in both extracts and all coffee types was lower than maltase and glucoamylase implying that sucrase inhibitory activity of all coffee types was higher than that of maltase and glucoamylase (Table 2). With the exception of glucoamylase, ethanol extracts of all coffee types showed higher inhibitory activity than DW extracts. The overall result revealed that in a dose-dependent manner the coffee extracts showed similar but significant inhibitory effects on sucrase, maltase, and glucoamylase. This suggested that the coffee extracts may be used as potential inhibitors of α-glucosidases with particular inhibitory effect on sucrase than maltase and glucoamylase.

**Table 2:** Half maximal inhibitory concentration (IC_50_) of coffee water (DW) extract and ethyl alcohol extract on rat small intestinal sucrase, maltase, and glucoamylase activity

		**IC**_**50**_** (mg/mL)**			
		**Sumatra**	**Guatemala**	**Ethiopia**	**Acarbose®**
**Sucrase**	DW	0.52	0.50	0.47	> 0.10
	Ethanol	0.43	0.39	0.41	
**Maltase**	DW	2.03	1.93	1.94	> 0.10
	Ethanol	1.60	1.67	1.83	
**Glucoamylase**	DW	1.11	1.01	1.08	> 0.10
	Ethanol	1.38	1.20	1.27	

Inhibition of sucrase, maltase, and glucoamylase plays an important role in controlling the rapid rise in blood glucose levels after excessive mixed carbohydrate meals. Therefore, limiting the amount of glucose/calories that can be absorbed by inhibiting the activity of α-glucosidases in the small intestine plays an important role in improving postprandial hyperglycemia.

### Blood glucose-lowering effect of coffee extracts *in-vivo*

**Fig. 4:**
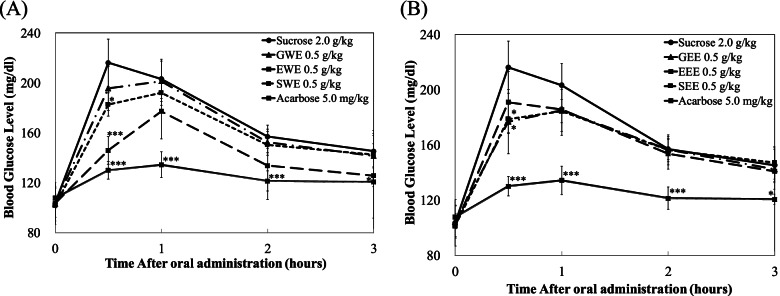
Effect of Coffee of water and ethyl alcohol extracts on sucrose loading test. After fasting for 24 h, 4-week-old, male SD rats were orally administered with sucrose (2.0 g/kg-body weight (bw)) with or without samples (GWE, EWE, SWE, GEE, EEE, SEE of 0.5 g/kg-bw, and Acarbose 5.0 mg/kg-bw). Each point represents mean ± S.D. (*n* = 5). *p < 0.05 and ***p < 0.001 compared to different samples at the same concentration by unpaired Student’s *t*-test

**Fig. 5:**
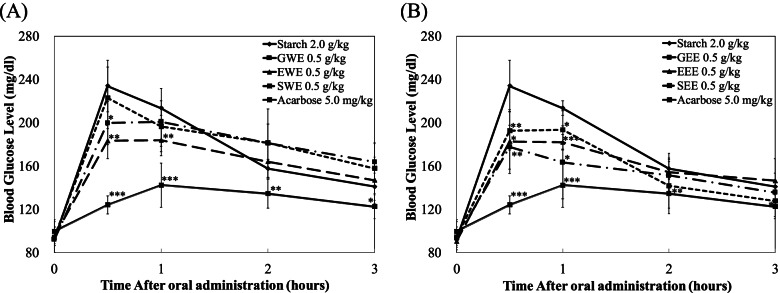
Effect of Coffee of water and ethyl alcohol extracts on starch loading test. After fasting for 24 h, 4-week-old, male SD rats were orally administered with starch (2.0 g/kg-body weight (bw)) with or without samples (GWE, EWE, SWE, GEE, EEE, SEE of 0.5 g/kg-bw, Acarbose 5.0 mg/kg-bw). Each point represents mean ± S.D.(n = 5). *p < 0.05, **p < 0.01 and ***p < 0.001 compared to different samples at the same concentration by unpaired Student’s *t*-test

To confirm the *in vitro* inhibition of sucrase activity, the time courses of plasma glycemic response were measured at 0, 30, 60, and 120 min after sucrose-loading (2.0 g/kg body weight (bw)) in SD rats. Since all the three coffee types showed similar inhibitory potential *in vitro* we assessed the blood-lowering effect of the three coffee types in *in-vivo*. Figures 4 and 5 show the comparison of the plasma glucose-lowering effect of water and ethanol extracts of Sumatra, Guatemala, and Ethiopia coffee types at the concentration of 0.5 g/Kg bw with the control group (sucrose or starch only) and a known pharmacological α-glucosidase inhibitor, acarbose (5 mg/kg bw) as a positive control.

At half an hour after sucrose loading, SWE and EWE significantly reduced plasma glucose level when compared to the control in SD rats (Fig. 4A). However, no significant plasma glucose-lowering effect was observed from GWE. On the other hand, EEE and GEE significantly decreased plasma glucose levels as compared to the control in SD rats at 30 min following sucrose loading (Fig. 4B).

When male SD rats were administered with starch solution EWE and SWE significantly decreased blood glucose levels (Fig. 5A). Meanwhile, SD rats treated with SEE, EEE, and GEE showed significant decreases in plasma glucose levels when compared to the control (starch). Acarbose, the well-known α-glucosidase inhibitor pharmacology therapy drug, showed significantly higher blood lowering than all the coffee types and the controls.

Both EWE and EEE showed significant blood lowering effect in sucrose and starch loading tests showing its potential as an α-glucosidase inhibitor (Fig. 5B). Overall, these results revealed the potential of both water and ethanol coffee extracts to reduce plasma glucose levels after a meal.

### Pharmacodynamics parameters

Pharmacodynamics (PD) parameters of the sucrose and starch loading tests with GWE, EWE, SWE and acarbose are shown in Table 3. Changes in PD parameters of control and after administration of GWE, EWE, SWE, and acarbose with sucrose or starch ingestions4. EWE-treated groups resulted significantly reduced C_*max*_ (Both sucrose and starch were *p* < 0.01) and AUC_*t*_ (sucrose was *p* < 0.05), however this reduction was less effective than the acarbose-treated group.

**Table 3:** Changes in pharmacodynamics (PD) parameters of control and after administration of GWE, EWE, SWE, and acarbose with sucrose or starch ingestions

Groups	**PD Parameters**		
	**C*max* (mg/dL)**	**T*max* (hr)**	**AUC*t* (hr·mg/dL)**
Sucrose 2.0 g/kg	222.3 ± 16.0	0.7 ± 0.3	516.0 ± 29.0
Acarbose 5.0 mg/kg	137.4 ± 8.3^***^	0.8 ± 0.3	376.2 ± 17.2^***^
GWE 0.5 g/kg	208.8 ± 14.4	0.8 ± 0.3	496.3 ± 23.9
EWE 0.5 g/kg	182.5 ± 15.4^**^	1.4 ± 0.9	434.8 ± 50.8^*^
SWE 0.5 g/lg	191.0 ± 9.1^**^	1.0 ± 0.0^*^	482.3 ± 12.7
Starch 2.0 g/kg	236.2 ± 25.1	0.5 ± 0.0	529.1 ± 13.4
Acarbose 5.0 mg/kg	144.1 ± 18.8^***^	1.3 ± 0.5^**^	389.0 ± 36.8^***^
GWE 0.5 g/kg	218.3 ± 20.6	1.0 ± 0.6	536.3 ± 49.4
EWE 0.5 g/kg	191.3 ± 13.2^**^	0.8 ± 0.3^*^	490.5 ± 34.6
SWE 0.5 g/lg	222.9 ± 30.1	0.5 ± 0.0	548.9 ± 47.2

The results were expressed as mean ± S.D. All parameters were compared between control and treatment group (GWE, EWE, SWE, and Acarbose) by unpaired Student’s *t*-test (*p < 0.05; **p < 0.01; and ***p < 0.001)

On the other hand, in Table 4. Changes in PD parameters of control and after administration of GEE, EEE, SEE and acarbose with sucrose or starch ingestions. All the ethanol extract coffee was shown significantly reduced C_*max*_ (GEE: *p* < 0.01, *p* < 0.05; EEE: *p* < 0.05, *p* < 0.01; SEE *p* < 0.01, *p* < 0.05). However, in terms of AUC*t* there was no significant difference among sucrose and all of ethanol extract coffee. Starch loading tests are shown GEE, EEE, and SEE significantly reduced AUC*t* (GEE: *p* < 0.01; EEE: *p* < 0.05; SEE *p* < 0.01) of blood glucose in rats ingested with starch compared to control.

**Table 4:** Changes in pharmacodynamics (PD) parameters of control and after administration of GEE, EEE, SEE, and acarbose with sucrose or starch ingestions

Groups	**PD Parameters**		
	**C**_***max***_** (mg/dL)**	**T**_***max***_** (hr)**	**AUC**_***t***_** (hr·mg/dL)**
Sucrose 2.0 g/kg	222.3 ± 16.0	0.7 ± 0.3	516.0 ± 29.0
Acarbose 5.0 mg/kg	137.4 ± 8.3^***^	0.8 ± 0.3	376.2 ± 17.2^***^
GEE 0.5 g/kg	188.7 ± 13.5^**^	0.8 ± 0.3	480.7 ± 34.1
EEE 0.5 g/kg	194.1 ± 18.3^*^	0.7 ± 0.3	483.6 ± 27.6
SEE 0.5 g/lg	185.9 ± 8.9^**^	0.9 ± 0.2	481.4 ± 11.8
Starch 2.0 g/kg	236.2 ± 25.1	0.5 ± 0.0	529.1 ± 13.4
Acarbose 5.0 mg/kg	144.1 ± 18.8^***^	1.3 ± 0.5^**^	389.0 ± 36.8^***^
GEE 0.5 g/kg	188.0 ± 25.7^*^	0.6 ± 0.2	454.9 ± 46.7^*^
EEE 0.5 g/kg	195.0 ± 13.9^**^	0.7 ± 0.3	477.8 ± 34.5^*^
SEE 0.5 g/lg	203.4 ± 14.5^*^	0.8 ± 0.3^*^	468.2 ± 37.0^**^

The results were expressed as mean ± S.D. All parameters were compared between control and treatment group (GEE, EEE, SEE, and Acarbose) by unpaired Student’s *t*-test (*p < 0.05; **p < 0.01; and ***p < 0.001)

## Conclusions

Non-insulin-dependent diabetes mellitus (NIDDM) is a metabolic disorder character-ized by hyperglycemia. It is a global health problem resulting to a lot of microvascular complications. Searching for natural drugs with minimum or no side effects is an attractive approach for the potential management of NIDDM. Our results revealed that coffee can be a source of phenolic compounds which can reduce postprandial blood glucose levels. Our data suggest that the use of coffee extracts can potentially be applied as inhibitors of α-glucosidases, exerting a similar mechanism with acarbose, for glycemic control.

We observed that the inhibitory activities did not correlate with the total phenolic contents. Previous reports have identified that the inhibition of carbohydrate hydrolyzing enzymes is not only dependent on phenolic content, but also on phenolic profile. Additionally, during roasting of coffee beans, secondary metabolites are produced (such as amadori compounds and melanoidins), that can exert inhibitory activity against carbohydrate hydrolyzing enzymes. When green coffee beans are roasted under high temperatures, chemical reactions between amino acids and carbohydrates, known as Maillard reactions, create a number of unique components. During roasting total phenolic contents in coffee are reported to be decreased significantly [12]. Therefore melanoidins, in addition to phenolics compounds might be contributed to the inhibitory effect of carbohydrate hydrolyzing enzymes.

It is interesting to notice that the tested coffee extracts did not exhibit and α-amylase inhibitory activity. Our *in vitro* and *in vivo* studies provide a biochemical rationale for further animal and clinical studies. However, further work is still needed to identify specific anti-hyperglycemic bioactive compounds in the coffee extracts and evaluate pharmacological and biological effects. Finally, in our case, we used only mild-roasted coffee but since phenolic contents in coffee can be affected by roasting degree and methods additional studies are needed using coffee extracts roasted by various degrees and methods.

## Data Availability

The datasets generated during and/or analyzed during the current study are available from the corresponding author on reasonable request.
